# A group brain-controlled method for UAVs using a hybrid paradigm of hand movements and visual evoked potentials

**DOI:** 10.3389/fnbot.2026.1858496

**Published:** 2026-08-21

**Authors:** Rui Li, Shikun Bian, Yaxi Deng, Xiaoqing Wei, Shiqiang Yang, Yang Liu, Jiangcheng Chen

**Affiliations:** 1 School of Mechanical Engineering, Xi'an University of Technology, Xi'an, China; 2 The School of Art and Design, Xi'an University of Technology, Xi'an, China; 3 Department of Data and Systems Engineering, Advanced Technologies Institute, The University of Hong Kong, Hong Kong, Hong Kong SAR, China

**Keywords:** brain computer interface, convolutional neural network, EEG signal decoding, hand movement, shared control

## Abstract

Brain–computer interfaces (BCIs) are among the most prominent communication technologies that establish a direct channel for information exchange between the brain and external devices. They have been extensively applied in the field of aerospace. However, traditional BCI technology faces challenges, including a limited number of brain control commands and insufficient recognition accuracy in electroencephalography (EEG) decoding. These limitations make it difficult for traditional BCIs to perform complex tasks with high accuracy. Therefore, this study proposed a novel group BCI (G-BCI) system and further constructed a brain–machine shared control method for unmanned aerial vehicle (UAV) swarm control. First, a novel G-BCI paradigm combining precise hand movements and visual evoked potentials was designed. Moreover, an improved multi-domain feature fusion convolutional neural network (MDFF-CNN) was employed to decode EEG and electromyography (EMG) signals from precise hand movements, while a Filter Bank Common Spatial Patterns with Canonical Correlation Analysis (FBCCA) method was used for Steady-State Visual Evoked Potentials (SSVEP) decoding. Furthermore, a task-driven shared control model mapping the G-BCI system and the leader–follower UAV swarm control strategy was proposed. To verify the effectiveness of the proposed method, eight participants were recruited to conduct both offline and online experiments. The proposed G-BCI system achieved an offline accuracy of 88.91 ± 5.06% and an online accuracy of 88.89 ± 1.96%. All the experimental results demonstrate the feasibility of the proposed method.

## 1 Introduction

A Brain–computer interface (BCI) is a cutting-edge technology that enables direct communication between the human brain and external devices by decoding electroencephalography (EEG) signals from the cerebral cortex and converting them into control commands for peripheral devices (Willsey et al., 2025; Tan et al., 2023). This technology allows users to control intelligent devices according to their own intentions in complex environments without relying on the peripheral nerve–muscle pathway (Tang et al., 2023; Zbinden et al., 2024; Simeral et al., 2021; Collinger et al., 2018). Although significant progress has been made in BCI technology, traditional BCI systems still face challenges such as limited brain commands, low EEG signal decoding accuracy, and poor generalization (Yang, 2024; Zhao et al., 2025; Liu et al., 2023). To overcome these shortcomings, a group brain–computer interface (G-BCI) approach involving multiple users has been proposed by scholars (Si et al., 2024; Jiang et al., 2019). This method employs the spatio-temporal complementarity of EEG signals from multiple users to construct a distributed neural decoding and collaborative decision-making framework. It effectively expands the dimensionality of brain control commands and enhances system robustness, thereby providing a theoretical basis for group control in complex tasks (He and Wu, 2020; Nagender et al., 2023). In recent years, G-BCI technology has gradually become a research hotspot. Tsinghua University and the University of California collaborated (Bhattacharyya et al., 2021) to develop an online collaborative BCI system that integrates the visual evoked potentials of multiple users to improve the detection speed and classification accuracy of visual targets. The classification accuracy of the proposed system reached over 90%, which was 11% higher than that of a single BCI. A team from the Korea Advanced Institute of Science and Technology (Yuan et al., 2013) developed a collaborative BCI system that improved decision speed and accuracy in visual Go/NoGo tasks by jointly analyzing the EEG signals from multiple users. The average classification accuracy in collaborative mode was 78.0%, which was 12.9% higher than that in the single-user mode. Touyama (2014) developed a collaborative novel G-BCI system based on P300 signals, enhancing performance, with online experiments achieving a recognition accuracy of 77.7%. When the number of users increased to nine, the accuracy rate rose to 80.3%. Moreover, Jiang et al. (Jiang et al., 2019) in the United States proposed a novel multiuser brain-to-brain interface system called BrainNet, which enabled direct brain-to-brain communication between three individuals by combining EEG signals with targeted wireless stimulation. The average task completion accuracy of this system reached 81.3%. In 2024, Professor Si et al. (Si et al., 2024) proposed a member selection strategy based on maximum individual capability and maximum collaborative capability (MIMC) to optimize the performance of collaborative BCI systems in rapid serial visual presentation (RSVP) tasks. In a two-person matching task, the MIMC strategy significantly improved the classification of the system while reducing the number of users involved and maintaining performance. All the above research indicates that G-BCI technology has effectively improved the decoding accuracy of EEG signals. However, several challenges remain, including insufficient control command dimensionality and low decoding accuracy.

The main drawback of existing G-BCI systems is their simplistic control paradigms, which severely restrict their ability to support complex control tasks. Furthermore, the traditional shared control methods are susceptible to low-quality EEG signals, leading to unstable classification performance during collaborative decision-making, particularly under high-dimensional UAV swarm control conditions. Therefore, enhancing the richness of control commands and the robustness of EEG decoding is crucial for realizing practical applications.

The primary objective of this study was to address classification accuracy and overcome the limitations of insufficient brain control modes.

In response to the aforementioned challenges, a G-BCI system with a hybrid paradigm has been proposed for complex motion control of UAV swarms. Unlike traditional single-user BCI control strategies that rely on EEG signals, this study proposes a novel hybrid G-BCI system that integrates EEG and EMG signals (Li and Wang, 2022). Furthermore, a multi-user collaborative framework for a shared control strategy was developed based on the G-BCI system and the leader–follower control strategy to support complex UAV swarm missions. The key contributions of this study derived from experimental validations are outlined as follows:

1) To solve the limitation of insufficient brain commands, a two-subject G-BCI system was constructed to increase the dimensionality and robustness of brain control commands. A hybrid paradigm integrating precise hand movements and visual evoked potentials was designed to expand the number of brain control commands.
2) To improve the unsatisfactory accuracy of EEG signals associated with precise hand movements, a decoding algorithm using an improved multi-domain feature fusion convolutional neural network (MDFF-CNN) integrating EEG and EMG signals was developed. Compared to conventional decoding methods, the proposed model can extract temporal and spatial features more robustly.
3) To further enhance the performance of the shared control strategy for UAV swarms, a task-driven shared control model mapping the G-BCI system and the leader–follower UAV swarm control strategy was developed for motion control. This method provides a novel strategy for complex swarm motion control, achieving better performance than traditional direct BCI models.

The remainder of this article is structured as follows: Section 2 describes the structure of the G-BCI system and provides an overview of the experimental process and the hybrid paradigm design. Section 3 describes the details of the decoding algorithm and introduces the shared control method proposed in this article. Section 4 presents the experimental results and analysis. Section 5 discusses the findings, and Section 6 concludes the article.

## 2 Experiment

### 2.1 Participants and group EEG signal recording

In this study, eight healthy participants aged from 22 to 30 years were recruited for both offline and online experiments. None of them had a history of health problems or prior experience with BCI experiments. Before the experiment, each participant signed a written informed consent form. The Institutional Review Board of Xi'an University of Technology approved the proposed study, and all experiments were conducted in accordance with the Declaration of Helsinki.

**Figure 1:**
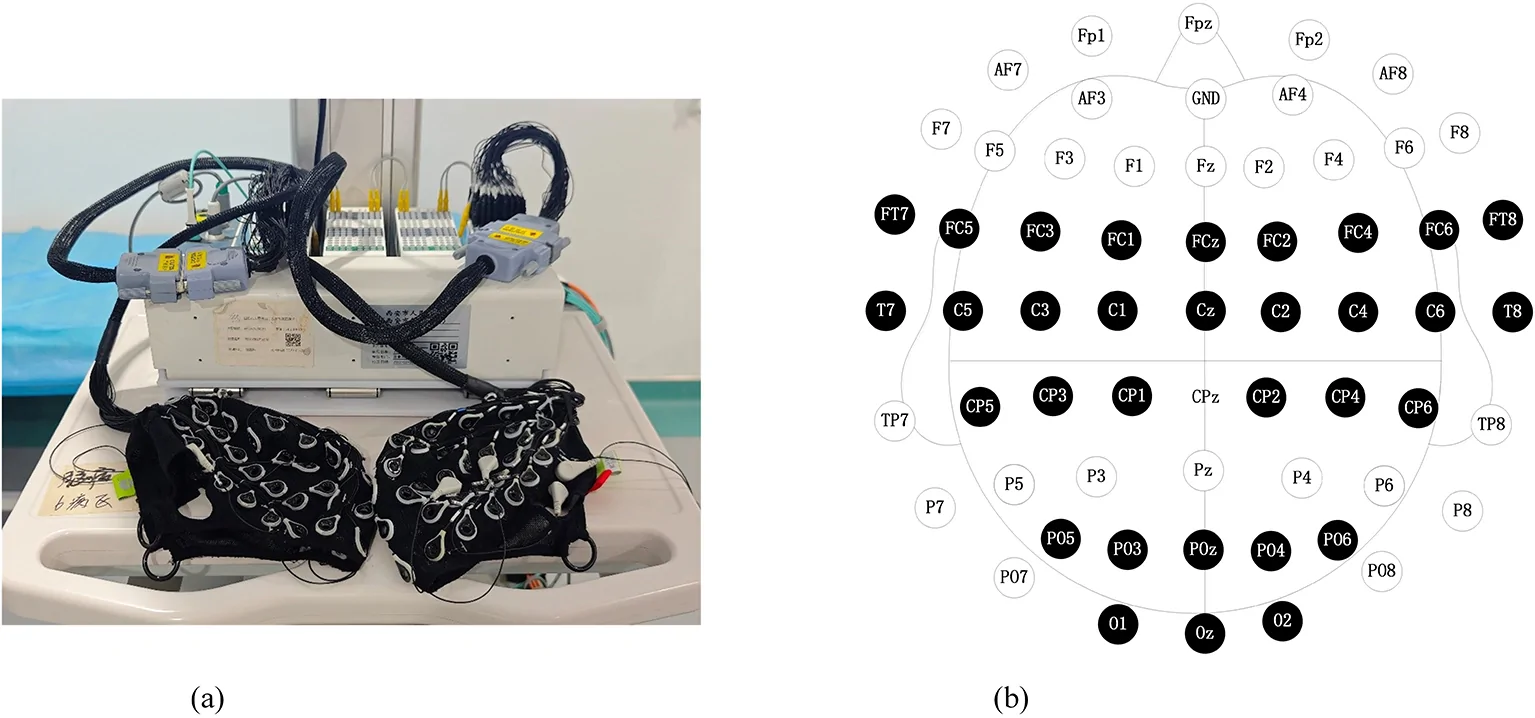
The group EEG recording system of NeuSen-NSH1024 and its channel location. **(a)** NeuSen-NSH1024. **(b)** The selected 32-channel location.

A NeuSen-NSH1024 system, shown in Figure 1a, was used to record group EEG data in this study. It is a high-precision EEG signal acquisition device that supports the simultaneous recording of 16 users, with 64 channels for each user. The channel distribution followed the international standard 10–20 electrode location system, and the sampling rate was set to 1,000 Hz. During the experiment, 32 channels were selected according to neurophysiological mechanisms. The following electrodes were selected from the motor cortex and occipital cortex, including FT7, FC5, FC3, FC1, FCz, FC2, FC4, FC6, FT8, T7, T8, C5, C3, C1, Cz, C2, C4, C6, CP5, CP3, CP1, CP2, CP4, CP6, POz, PO3, PO4, PO5, PO6, Oz, O1, and O2. AFz and CPz served as the reference and ground electrodes, respectively. The electrode distribution and the location of the selected channels are provided in Figure 1b. During the experiment, the impedance of all electrodes was maintained below 10 KΩ.

### 2.2 G-BCI system for UAV swarm control

**Figure 2:**
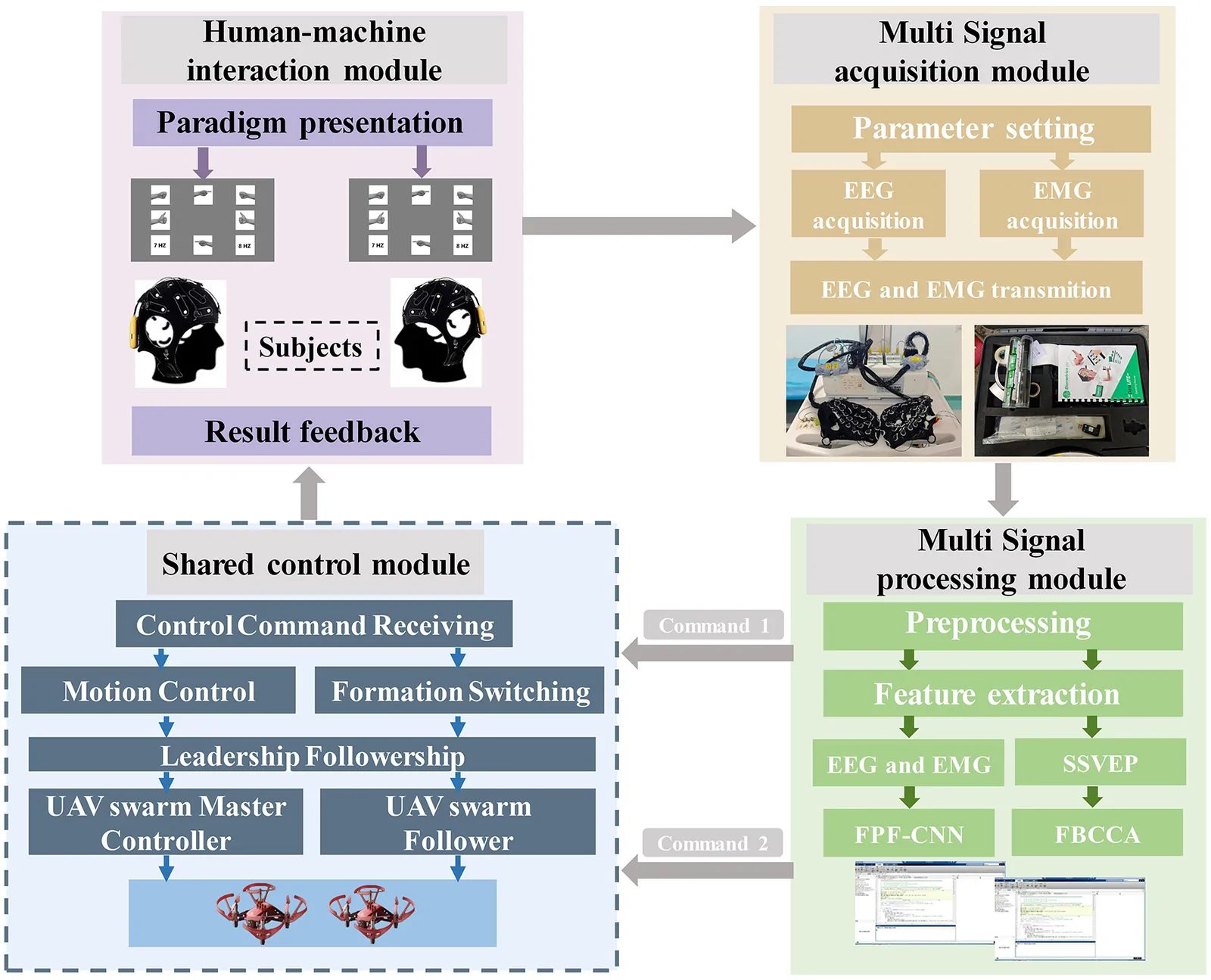
The framework of group BCI system.

The overall architecture of the proposed G-BCI system for UAV swarm control is shown in Figure 2. It consisted of a human–machine interaction module, a multi-signal acquisition module, a signal-processing module, and a shared control module. All modules of the G-BCI system used wireless communication technology to transmit information through Xiaomi R2P routers.

When the system was working, the human–computer interaction module employed a Lenovo Legion Y9000 laptop and a 24-inch Hanshida external monitor to present the hybrid G-BCI paradigms. At the same time, group EEG and EMG signals were recorded using a NeuSen-NSH1024 system developed by Neuracle and a DataLOG portable EMG recording device, respectively. Then, multi-neural signals were decoded by the multi-signal processing module using the same Lenovo Legion Y9000 laptop. After that, the decoding results obtained from the signal processing module were transmitted to the shared control module to control UAV swarms. In total, two DJI Robomaster TT quadcopters with secondary development capabilities were employed as the UAV swarms. The detailed parameters of the UAV swarms are provided in Table 1.

**Table 1:** Parameters of the UAV system.

RoboMaster TT unmanned aerial vehicle (Model TLW004)	
Takeoff weight	87 g
Maximum level flight speed	28.8 km/h
Maximum flight time	13 min (windless conditions)
Operating temperature range	0–40 °C
Operating frequency	2.4–2.4835 GHz

### 2.3 Hybrid brain control paradigm design for UAV swarm control

To improve the robustness and safety of UAV swarms during execution, this study proposed a hybrid paradigm based on the combination of precise hand movement execution and SSVEPs. The hand movement execution in the hybrid brain control paradigm included six kinds of left- and right-hand movements: Palmar grasp of the right hand (PGR), pointing with the right hand (PR), thumbs-up with the right hand (TR), palmar grasp of the left hand (PGL), pointing with the left hand (PL), and thumbs-up with the left hand (TL). The SSVEP stimulus unit used a basic black-and-white reversal stimulation pattern. The mathematical model of the SSVEP paradigm presentation can be described as follows:


$$\begin{array}{l} F=\left|P-\frac{255}{2}\left(1+\sin\left(2\pi f\times \frac{n}{f_{s}}\right)\right)\right|n=1,2,\cdots \;,N \end{array}$$ (1)

Where *F* is the matrix of pixel values of a stimulus at a given moment in time; *P* denotes the matrix of pixel values of scene images; *f* is the sinusoidal modulation frequency; *fs* represents the screen refresh rate of the computer, in this study the screen refresh rate was 60 Hz; *n* is the frame rate variable; and *N* is the total number of flickering frames.

Considering the safety and stability of UAVs during actual flight, the brain commands from six precise hand movements were employed to control the direction and formation changes of the UVA swarm, while SSVEP commands served as a safety command to govern the take-off and landing of the swarm.

**Figure 3:**
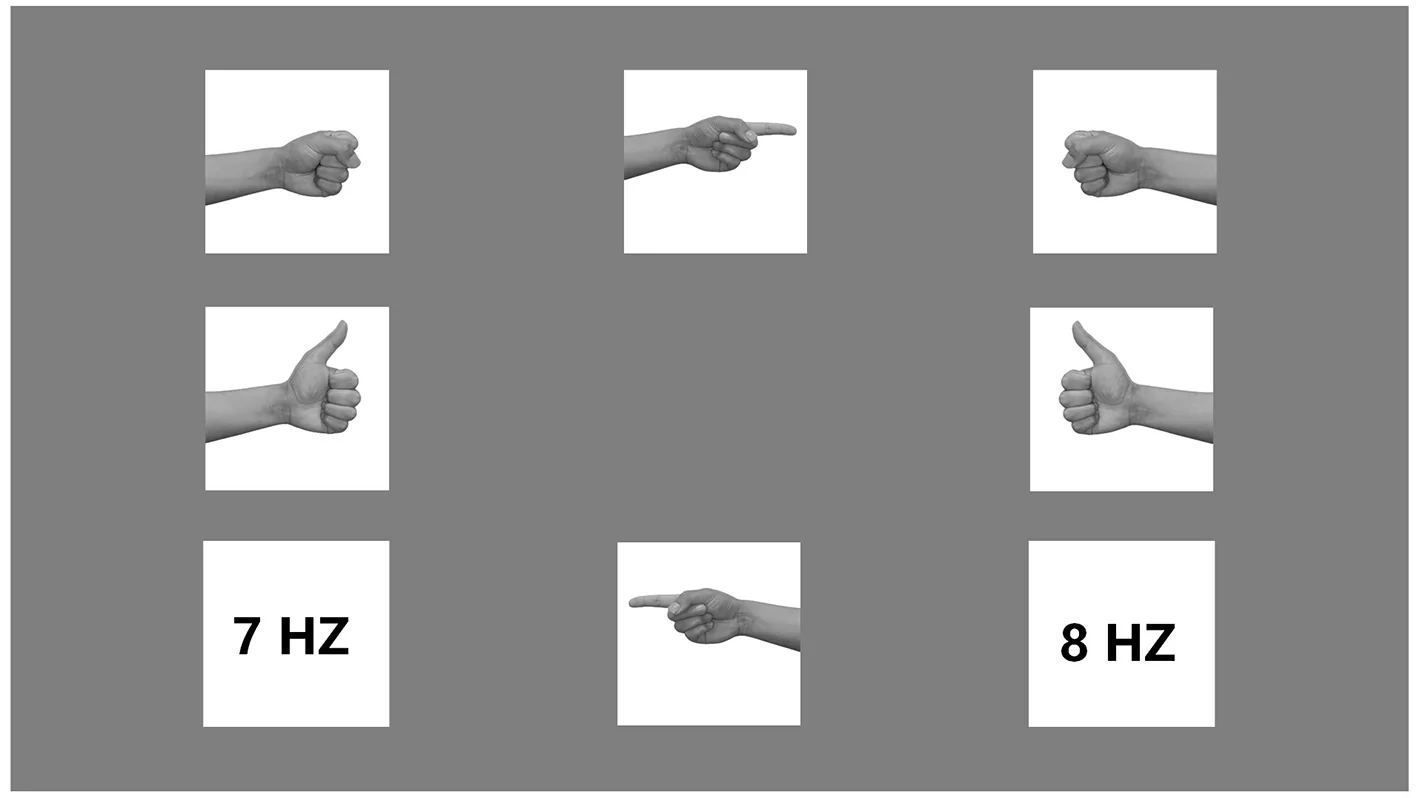
The hybrid paradigm with hand movement and SSVEP.

To facilitate SSVEP identification, the two stimulation frequencies were set to 7 and 8 Hz, respectively. Moreover, MATLAB's Psychtoolbox was used to implement the presentation of the hybrid paradigm. The final realized interface of the hybrid brain control paradigm is shown in Figure 3.

### 2.4 Experimental procedure

#### 2.4.1 Offline experiment

The experiment comprised two parts. The offline experiment evaluated the efficiency of the proposed hybrid paradigm. The online experiment investigated the feasibility of the proposed G-BCI system for controlling UAV swarms.

**Figure 4:**
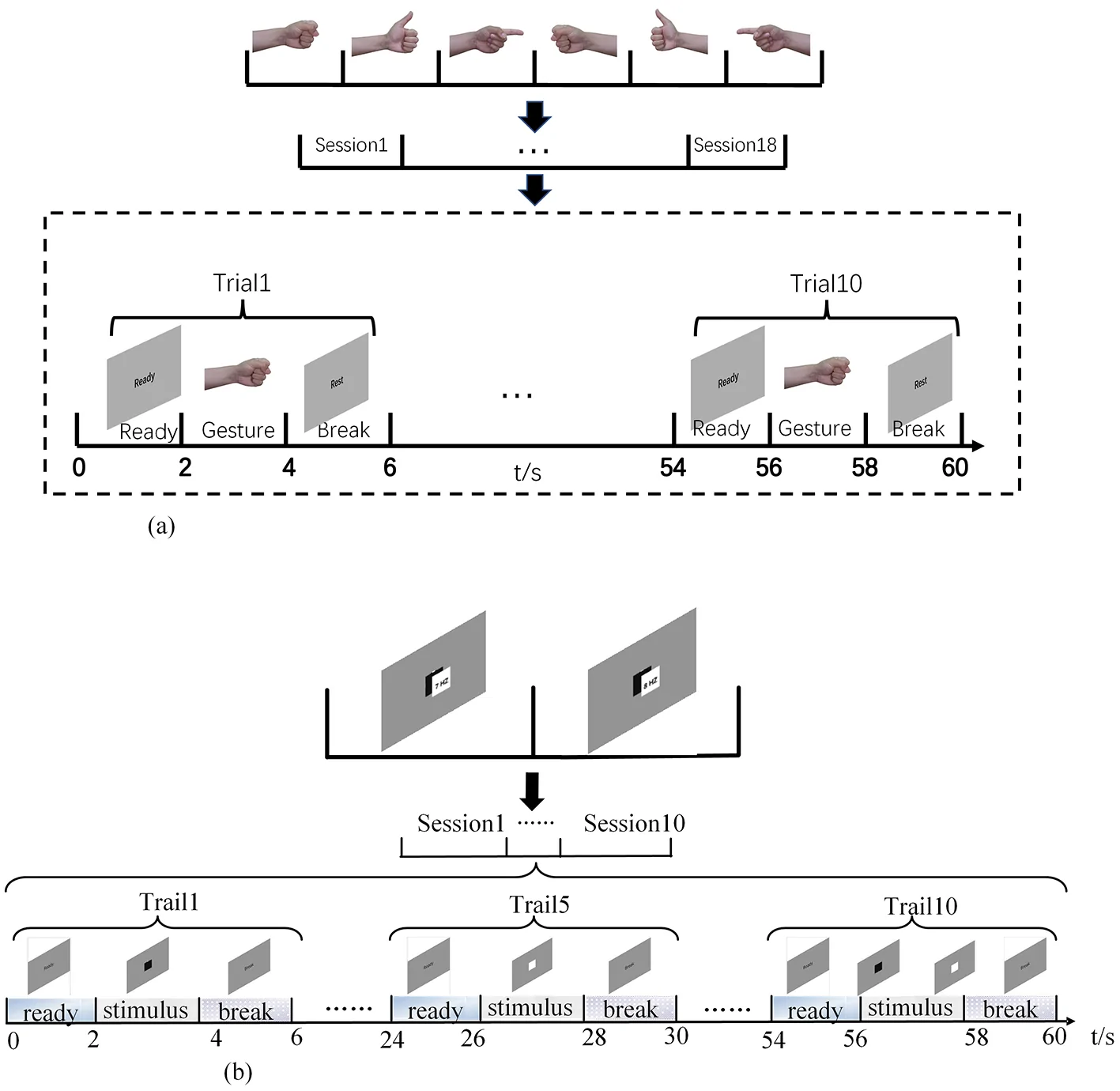
The offline experimental procedure. **(a)** The offline experimental procedure of the hand movement paradigm. **(b)** The offline experimental procedure of SSVEP paradigm.

During the offline experiments, EEG signals induced by different hand movements and SSVEP responses were acquired for 10 sessions, and each session contained 10 trials. Each trial consisted of a 2 s preparation period, a 2 s hand movement execution period, and a 2 s rest period. To avoid fatigue, a 1–2 min rest interval was provided between sessions, and a 5–10 min rest interval was provided between different gesture movements. The offline experimental process of the hybrid paradigm is shown in Figure 4.

#### 2.4.2 Online experiment

The online experiments were conducted in an indoor working environment, where the experimental platform was constructed. The single-trial time arrangement of the online experiments was consistent with that of the offline experiments. The mapping relationship between the brain control commands and the UAV swarm states in the online experiment is shown in Table 2.

**Table 2:** Precise hand movements vs. UAV brain control commands.

Precise hand movements	Control command 1	Control command 2
PGL	Up	Horizontal queue
PGR	Down	X-queue
TL	Turn left	Vertical queue
PR	Backward	T-queue
PL	Forward	V-queue
TR	Turn right	W-queue
Visual evoked stimuli unit 1	Landing safely	Initial queue
Visual evoked stimuli unit 2	Hover	Holding queue

The online experiment aimed to enable a complete set of flight tasks by UAV swarms, and the procedure for online motion control was as follows:

Step 1: Two participants, S1 and S2, simultaneously performed a PGL hand movement when the hybrid paradigm was initiated, and the UAV swarm performed an upward flight mission while maintaining a horizontal formation.

Step 2: Two participants, S1 and S2, performed their PL hand movements simultaneously when the brain control paradigm was initiated, and the UAV swarms switched their flight formation from the horizontal queue to the vertical queue after performing the forward flight mission.

Step 3: Participant S1 performed the TL hand movement, and participant S2 performed the PL hand movement at the start of the paradigm, and the UAV swarm changed its formation from a horizontal queue to a V-shaped formation after performing a forward flight mission.

Step 4: Participant S1 performed the PL hand movement when the brain control paradigm was initiated, while participant S2 did not perform any action, so the UAV swarm performed a right turn while maintaining the V-shaped formation in place.

Step 5: Participants S1 and S2 simultaneously gazed at visual stimulus 1. The UAV swarm landed while maintaining the current formation, and the whole experiment was completed.

In the online experiment, each group of participants completed three sessions, with each session containing six trials.

**Figure 5:**
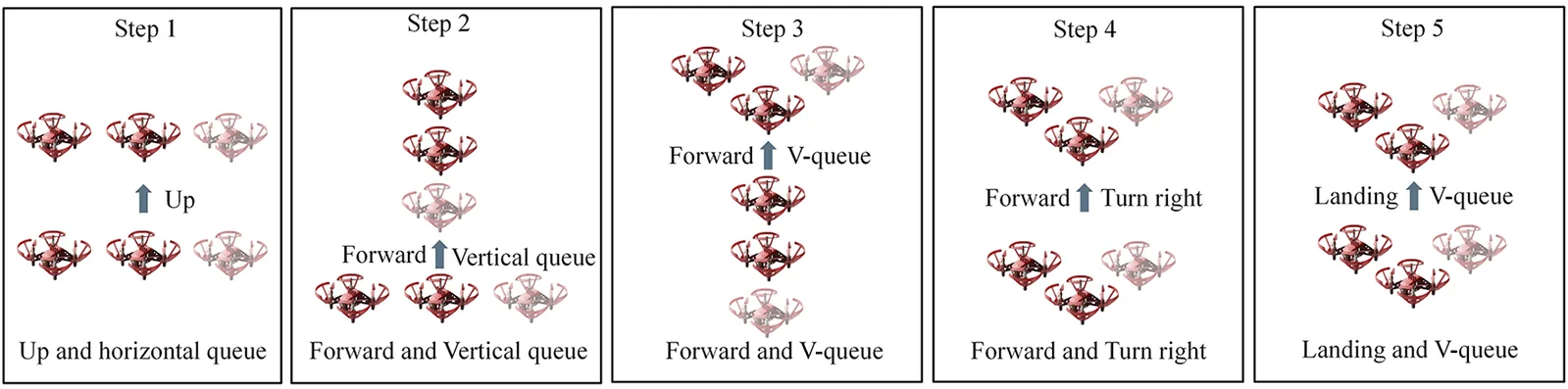
The procedure of online motion control for UAV swarms.

The online motion control process of the UAV swarm is shown in Figure 5.

## 3 Methods

### 3.1 Decoding algorithms

Since convolutional neural networks can automatically learn appropriate features from EEG and EMG data, they have been widely used in neural signal decoding (Lv et al., 2024). Hence, this study selected the MDFF-CNN algorithm based on multimodal signal fusion to jointly decode EEG and EMG signals associated with hand movements.

#### 3.1.1 EEG signal decoding algorithm of the MDFF-CNN model

This study employed an MDFF-CNN model that fused EEG signal features from different domains and EMG features associated with precise hand movements. After extracting multi-domain features from EEG and EMG signals using CNNs, these features were fused through weighted summation to decode the participants' movement intentions. Specifically, the MDFF-CNN model consists of an EEG time-domain feature convolutional submodule, an EEG frequency-domain feature convolutional submodule, an EEG spatial-domain feature convolutional submodule, and an EMG feature convolutional submodule.

The basic structure of the MDFF-CNN convolutional submodule includes a convolutional layer, a batch normalization (BN) layer, a pooling layer, a flatten layer, a concatenate layer, and finally a softmax activation function to perform single-domain feature-based hand movement intention recognition. The network parameters of each layer in the convolutional submodule were set as follows: the length of the input feature vector was determined by the dimensionality of the corresponding feature type. Furthermore, two one-dimensional (1D) convolutional layers were used for adaptive feature extraction from multimodal features. The first 1D convolutional layer had two filters with a kernel size of 3 × 1; the second 1D convolutional layer had eight filters, and the kernel size was set to 3 × 1. Then, the network convergence speed was improved using a BN layer. The output features from the convolutional layers were converted into one-dimensional vectors using a flatten layer, and intent recognition was performed based on single-domain features.

**Figure 6:**
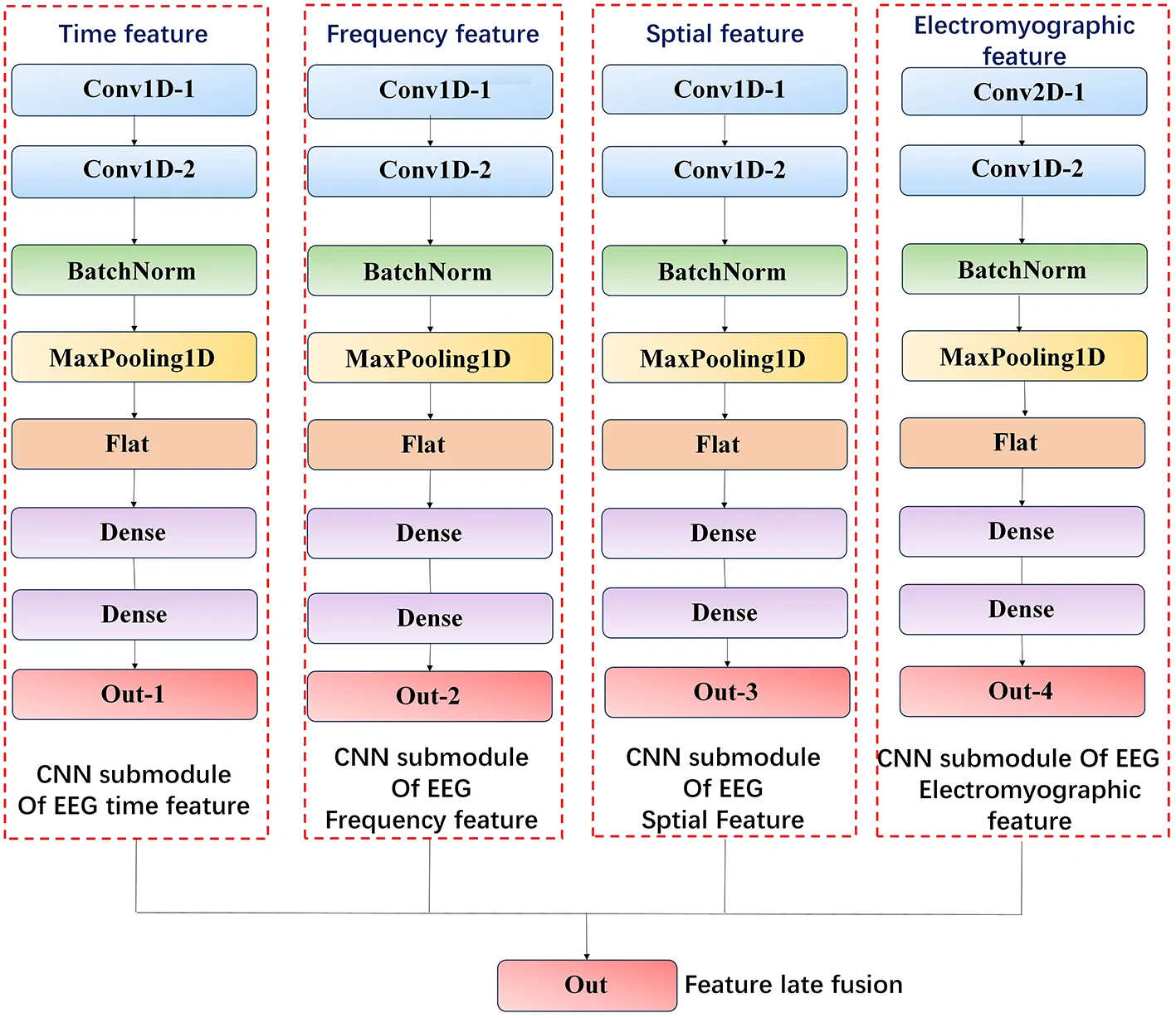
The Algorithm model of MDFF-CNN.

An output layer with a softmax activation function was added after the fully connected dense layer in each single-domain convolution submodule to achieve classification of precise hand movements based on single-domain features using EEG and EMG signals. The output results of each convolutional submodule were integrated using result weighting to obtain the final precise hand movement classification results based on the MDFF-CNN model. Considering the importance of features from EMG and EEG signals across different domains, the weights assigned to each type of feature were equal, meaning *w*_1_ = *w*_2_ = *w*_3_ = *w*_4_ = 0.25. The algorithm model structure based on the MDFF-CNN model is shown in Figure 6.

The remaining parameters of the MDFF-CNN algorithm were set as follows: The dropout rate was set to 0.5, the batch size was set to 32, the number of epochs was set to 64, and the number of iterations was set to 200. The cross-entropy loss function was used to calculate the difference between the predicted values and the true labels during MDFF-CNN training. The Adam optimizer was used to update the parameters, thereby obtaining the optimal training result.

The time-domain features of EEG signals induced by precise hand movements included the average amplitude, skewness, kurtosis, minimum negative peak value, maximum difference, absolute area, standard deviation, and slope of EEG signals from the 24 channels of the sensorimotor cortex. The specific calculation formula is shown in Table 3.

**Table 3:** Time-domain features extracted from EEG signals.

Features	Formula
Amplitude average value	$\mu =\frac{1}{N}\sum_{i=1}^{N}x_{i}$
Skewness	S=1N∑i=1N(xi−μ)3(1N−1(xi−μ)2)32
Kurtosis	$K=\frac{\frac{1}{N}\sum_{i=1}^{N}{\left(x_{i}-\mu \right)}^{4}}{{\left(\frac{1}{N-1}{\left(x_{i}-\mu \right)}^{2}\right)}^{2}}$
Minimum negative peak	*negative*_*peak* = min(*X*)
Maximum difference	*peak*_*to*_*peak* = max(*X*) − min(*X*)
Absolute area	$simps=\int_{a}^{b}|f(x)|dx$
Standard deviation	$\sigma =\sqrt{\frac{1}{N}\sum_{i=1}^{N}{\left(x_{i}-\mu \right)}^{2}}$
Slope	$slope=\frac{X}{T}$

In addition, the frequency-domain features of EEG signals induced by precise hand movements were extracted from the power spectral energy of the δ, θ, α, β, and γ waves across the selected 24 EEG channels from the motor cortex. The spatial-domain features of EEG signals induced by precise hand movements were extracted using the CSP algorithm with six spatial filters. Since each spatial filter can extract eight features, a total of 48 spatial-domain features were extracted. The EMG features associated with precise hand movements comprised five time-domain features, including the mean absolute error, root mean square value, waveform length, and zero-crossing rate, as well as two frequency-domain features, namely the average power frequency and median frequency. The dimensionality of the input features of each submodule of the MDFF-CNN model is shown in Table 4.

**Table 4:** Input feature dimensions of the MDFF-CNN model.

Input feature	Input dimension	Meaning
Time-domain feature	192 × 1	Eight time-domain features of 24 EEG signal channels
Frequency-domain feature	120 × 1	Five frequency-domain features of 24 EEG signal channels
Spatial-domain feature	48 × 1	Six spatial filters, each extracting eight spatial-domain features
EMG features	28 × 1	Four-channel EMG signal time-domain features

The MDFF-CNN model employed a post-fusion feature fusion strategy, fully extracting the time-, frequency-, and spatial-domain features of EEG signals and fusing them with the features of EMG. Since EEG signals from precise hand movements are a type of spontaneous EEG, fully extracting their multi-domain features can effectively improve the classification accuracy of EEG signals from different hand movements.

To further estimate the effectiveness of the proposed MDFF-CNN algorithm, the conventional frequency-domain convolutional neural network (FD-CNN) was employed as a comparison algorithm. The structure and functional parameters of the FD-CNN algorithm were the same as those of the CNN submodule in the MDFF-CNN model. The input of the FD-CNN algorithm consisted of the power spectral energy features of the δ, θ, α, β, and γ waves extracted from 24-channel EEG signals from the motor cortex.

#### 3.1.2 SSVEP decoding algorithm

In this study, the Filter Bank Common Spatial Patterns with Canonical Correlation Analysis (FBCCA) method was used to analyze the SSVEP responses evoked by the hybrid paradigm. The core of the FBCCA method is the same as that of the CCA decoding algorithm (Shao et al., 2025). CCA is a multivariate statistical method used to measure the linear correlation between two sets of signals. Assuming two sets of signals, *X* and *Y*, CCA aims to find two linearly combined variables, *W*_*X*_ and *W*_*Y*_, such that the correlation coefficient between $x=X^{T}W_{X}$ and $y=Y^{T}W_{Y}$ is maximized. The correlation calculation process is as follows:


$$\begin{array}{l} \rho =\underset{w_{x},w_{y}}{\max}\frac{E\left(W_{X}^{T}XY^{T}W_{Y}\right)}{\sqrt{E\left(W_{X}^{T}XX^{T}W_{Y}\right)E\left(W_{X}^{T}YY^{T}W_{Y}\right)}} \end{array}$$ (2)

Where *ρ* is the maximized correlation coefficient; *X* denotes multi-channel SSVEP data; *Y* is the reference signal; and *W*_*X*_ and *W*_*Y*_ are the weight vectors from SSVEP data and reference data, respectively.

The typical correlation coefficients between the multi-channel EEG signals and the reference signals corresponding to the *K* stimulus frequencies were calculated, resulting in *K* correlation coefficients *ρ*_*i*_ (i = 1, 2, …, *K*). The frequency corresponding to the maximum correlation coefficient was identified as the response frequency corresponding to the EEG signal. The formula can be described as follows:


$$\begin{array}{l} c=\underset{f_{i}}{\max}\rho_{i},i=1,2,\ldots ,K \end{array}$$ (3)

To evaluate the effectiveness of the proposed G-BCI approach for UAV swarm control, accuracy and Cohen's kappa coefficient were used as performance metrics. A paired *t*-test was conducted to compare the classification accuracy and corresponding information transfer rate (ITR) between the FD-CNN and MDFF-CNN models. Following prior studies, the desired statistical power was set to 0.8 (1 – *β* = 0.8), and the significance level was set to *α* = 0.05.

### 3.2 Shared control method

**Figure 7:**
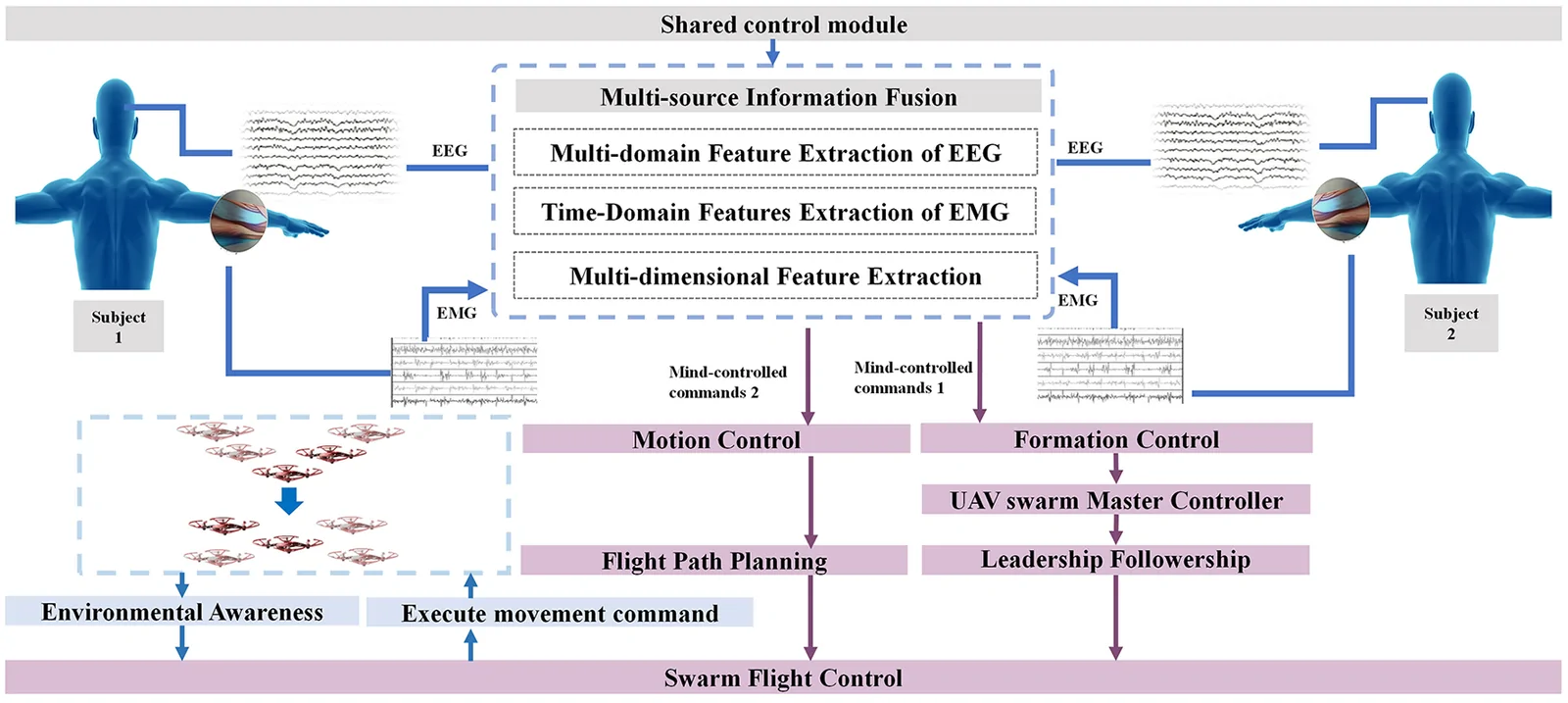
Brain-computer fusion shared control system for UAV swarms.

The brain–machine shared control method for UAV swarms combined the brain control commands of two participants with lightweight UAVs equipped with autonomous perception and navigation capabilities to achieve complex flight tasks. Figure 7 shows the structure of the proposed shared control method. Its structure mainly included the brain control module, the shared control module, and the UAV swarm control module.

Among these, the brain control module adopted the hybrid paradigm-based G-BCI system designed above, while the UAV swarm control module employed the leader–follower method as the control strategy after receiving the group brain-controlled commands.

By combining the hybrid brain control method with the leader–follower flight strategy for UAV swarms, a task-level control method for UAV swarms was realized. During the entire control process, the brain control intentions of participants S1 and S2 were used to control different flight states of the UAV swarms, with the specific intention commands shown in Table 2. SSVEPs were used to ensure the safe landing of UAV swarms. During system operation, the EEG signal recording device simultaneously collected EEG signals from the cerebral cortex of two participants and transmitted them to the pre-trained MDFF-CNN and FBCCA classifiers for decoding the participant's intentions. After two types of task-level brain control commands were sent to the UAV swarms, the two DJI RoboMaster TT quadcopters used their own flight planning algorithms to perform formation flight based on the brain control commands of the two participants.

## 4 Results

In this study, both offline and online experiments were conducted. The offline experiment was used to verify the effectiveness of the proposed hybrid paradigm and the MDFF-CNN algorithm. The online experiment was used to validate the feasibility of the proposed G-BCI approach for UAV swarm control.

### 4.1 Effectiveness of the proposed hybrid brain control paradigm

**Figure 8:**
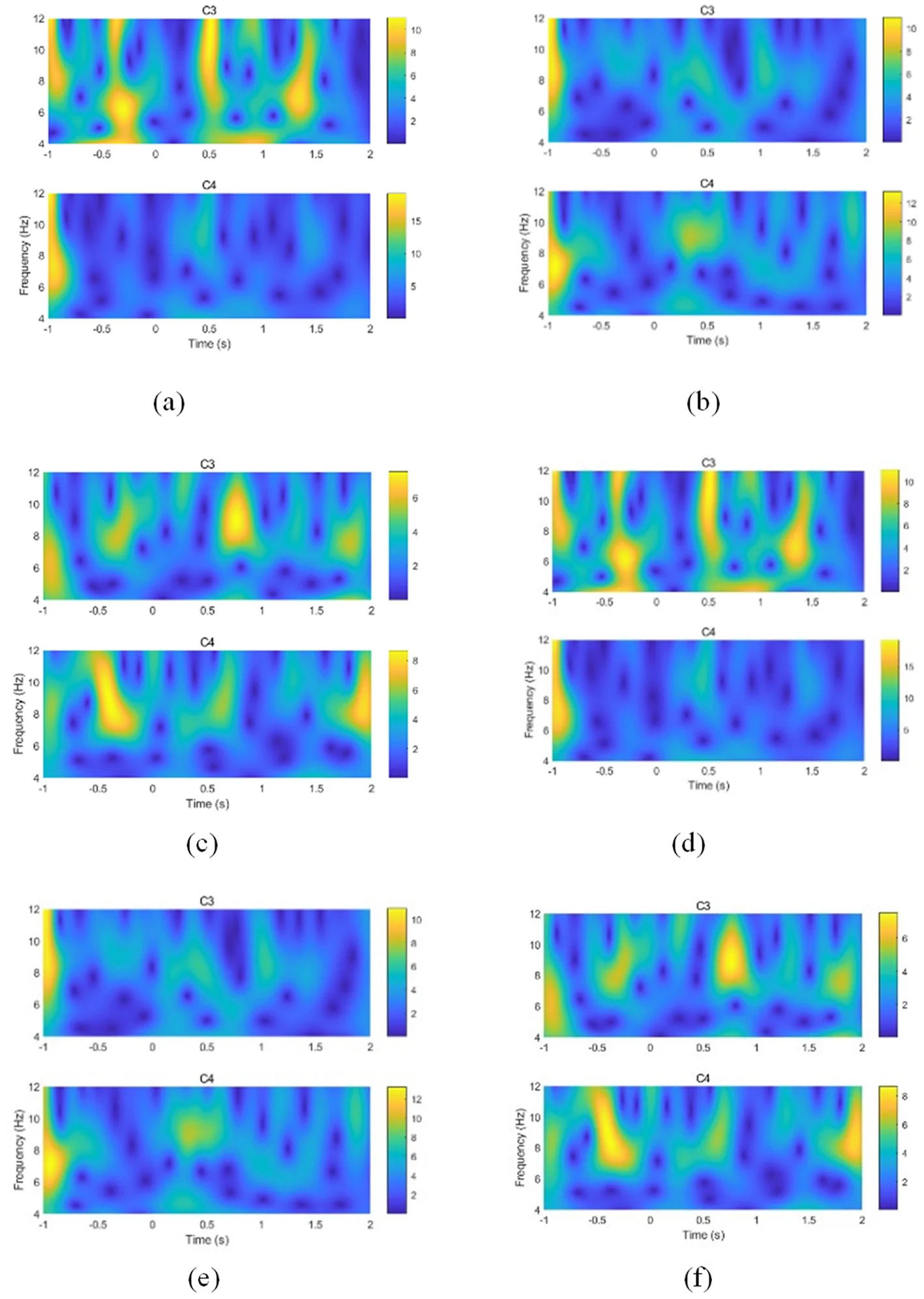
The time-frequency responses from EEG of six hand movements execution. **(a)** PGR. **(b)** PR. **(c)** TR. **(d)** PGL. **(e)** PL. **(f)** TL.

To confirm the differences in EEG signals from six types of hand movements, the continuous wavelet transform was used to analyze the time–frequency characteristics of the six types of precise hand movements. The EEG signals from each trial over the motion-related cortical area were averaged, and the brain response from one of the most representative participants, S3, is shown in Figure 8. It is evident that significant differences existed in the time–frequency characteristics of EEG signals from six different hand movements.

**Figure 9:**
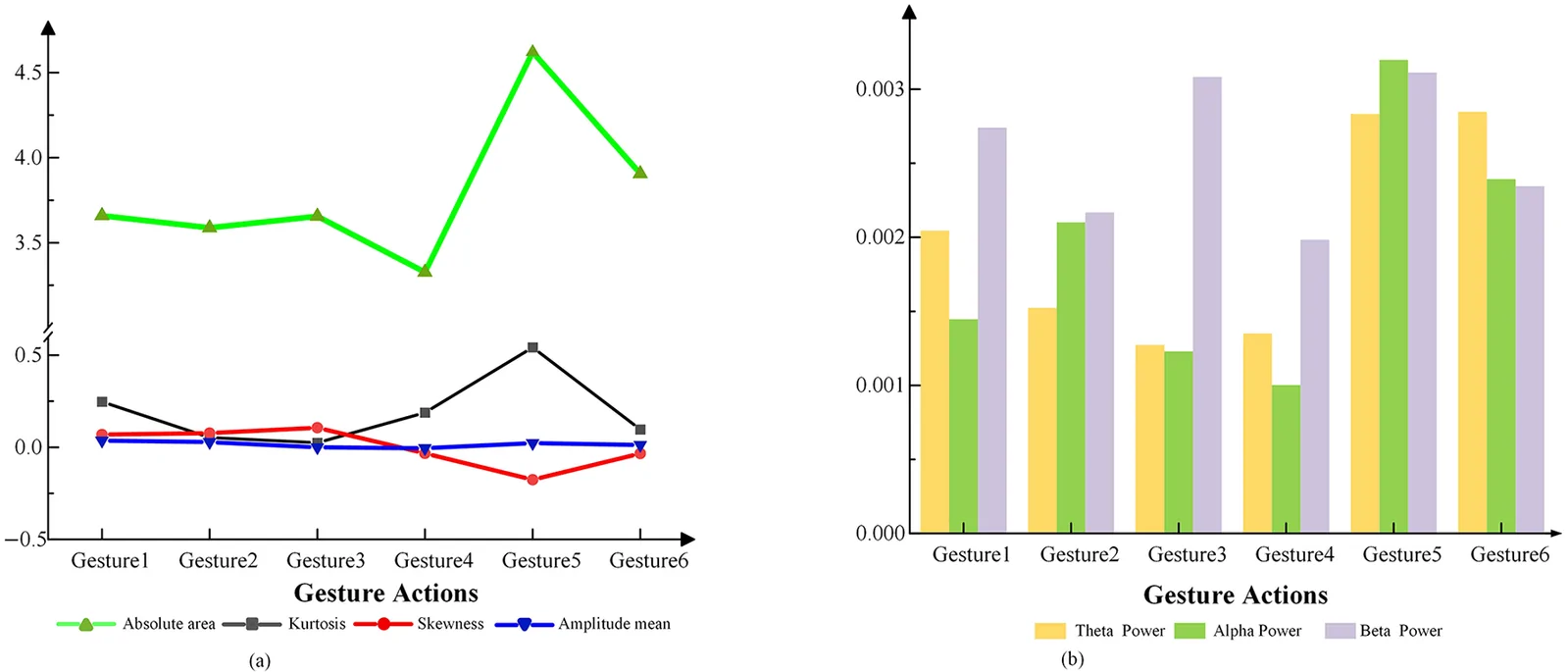
Analysis of the temporal features and frequency domain features of gestures actions. **(a)** Analysis of the temporal features. **(b)** Analysis of frequency domain features.

To verify whether time-domain features extracted from each precise hand movement exhibited differences, time-domain feature values from 24 channels over the motor cortex under each precise hand movement condition of the representative participant S3 were selected for analysis. The mean values of these channel features were calculated. Analysis was conducted on the time-domain characteristics extracted from the EEG signals associated with precise hand movements, including skewness, kurtosis, absolute area, and mean amplitude. The results are presented in Figure 9a.

Figure 9a presents the analysis results of the time-domain kurtosis, skewness, absolute area, and mean amplitude characteristics for six precise hand movements performed by the representative participant S3. The results revealed that certain differences existed in the time-domain features, including kurtosis, skewness, absolute area, and mean amplitude, among the six types of precise hand movements. Specifically, the absolute area demonstrated marked variations among the six precise hand movement patterns, with the greatest difference observed between movements 4 and 5. This indicates that the time-domain characteristics of EEG signals associated with precise hand movements are distinguishable. However, due to the limited discriminative ability of time-domain features, the six types of precise hand movement EEG signals cannot be effectively classified based solely on time-domain information.

To verify whether the frequency-domain features extracted from each precise hand movement exhibited differences, we selected the power spectral characteristics of 24 motor area channels from participant S3, a representative participant, during each precise hand movement for analysis. The features of these channels were averaged. The power spectral characteristics of three common frequency bands extracted from the EEG signals associated with precise hand movements were analyzed, with the results presented in Figure 9b.

Figure 9b demonstrates that the power spectral characteristics within the theta, alpha, and beta frequency bands exhibited certain differences across the six types of precise hand movements. Notably, the power spectral characteristics within the alpha frequency band revealed marked differences in the feature values of the six precise hand movements, with the greatest disparity observed between precise hand movements 4 and 5. This indicates that the frequency-domain characteristics of EEG signals associated with precise hand movements are distinguishable.

**Figure 10:**
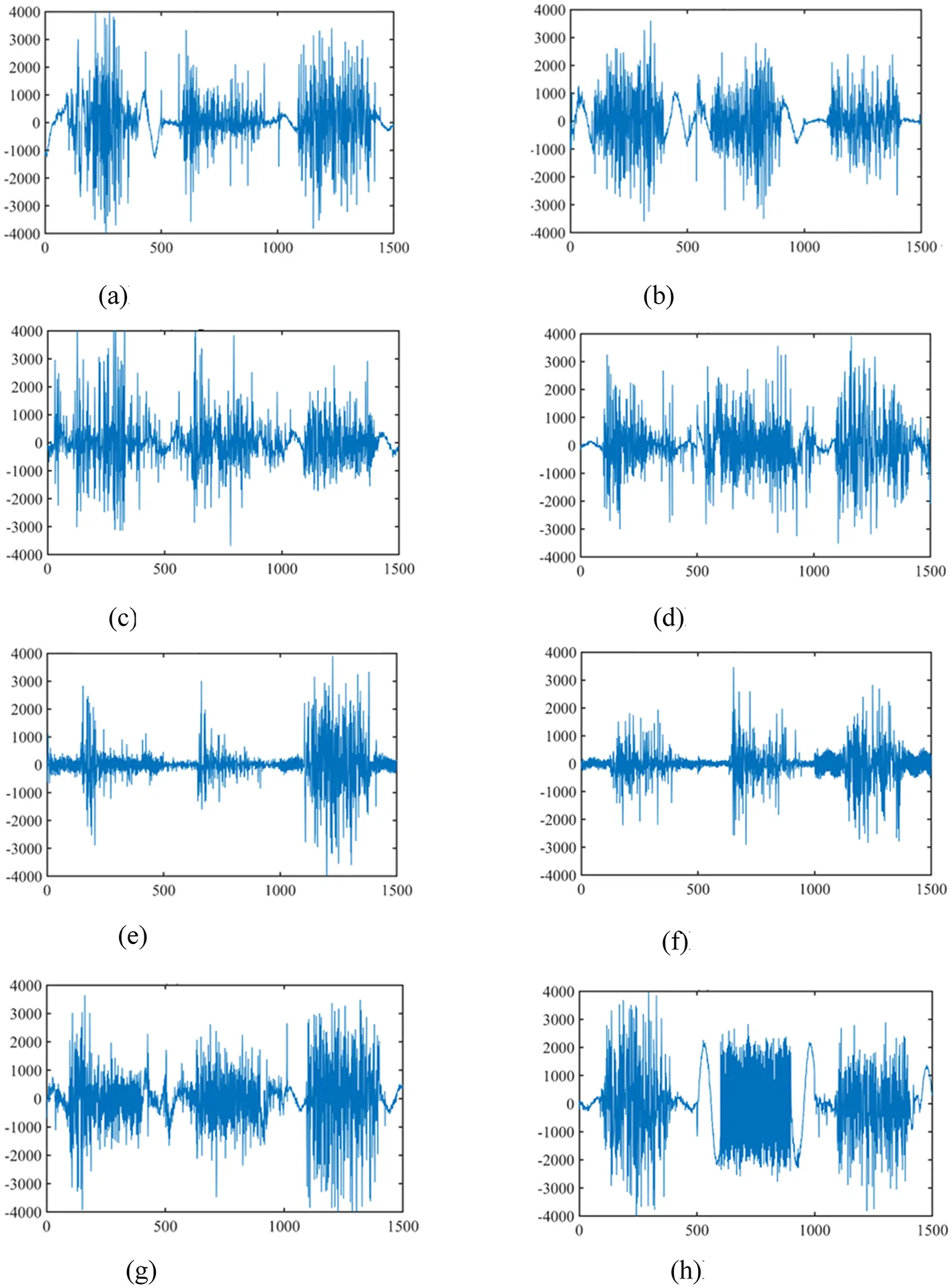
The time analyse results from EMG of two hand. **(a)** Right channel 1. **(b)** Right channel 2. **(c)** Right channel 3. **(d)** Right channel 4. **(e)** Left channel 1. **(f)** Left channel 2. **(g)** Left channel 3. **(h)** Left channel 4.

To further verify the validity of the proposed paradigm, the EMG responses associated with the six precise hand movements from all participants were analyzed, and certain regular and similar response patterns were observed. The EMG responses of one representative participant, S3, are displayed in this study. The EMG signals were first processed using a 20–200 Hz bandpass filter and a 50 Hz notch filter. The time-domain response characteristics of the representative palmar grasp movements of the left and right hands are shown in Figure 10.

The analysis results showed that the EMG responses induced by different precise hand movements exhibited typical variability. Overall, these findings confirm the effectiveness of the proposed hybrid paradigm.

### 4.2 Algorithm analysis

To further determine the effectiveness of the proposed MDFF-CNN method, the classification accuracy of eight participants was computed. The traditional FD-CNN model was employed as the comparison method, and the offline accuracy values of the two algorithms are listed in Table 5. The global accuracy of the proposed MDFF-CNN method was 79.20 ± 10.7%, and the corresponding kappa value was 0.75 ± 0.1. However, the averaged accuracy of the FD-CNN model was 74.02 ± 12.4%, and the corresponding kappa value was 0.60 ± 0.1. Compared to the conventional algorithm, the averaged accuracy increased by 5%, while its standard deviation decreased by 1.7%, with the highest accuracy reaching 89.42%. The classification results demonstrated the superiority of the MDFF-CNN decoding algorithm. Specifically, S5 had the lowest accuracy of 55.65%, which may have been affected by mental factors, such as fatigue and distractions during the experiments. The SSVEP classification results from FBCCA are listed in Table 6.

**Table 5:** The classification accuracy of the FD-CNN and MDFF-CNN models.

Participant	FD-CNN		MDFF-CNN	
	ACC (%)	Kappa	ACC (%)	Kappa
S1	70.60	0.62	73.51	0.68
S2	57.07	0.69	79.5	0.75
S3	88.65	0.52	89.42	0.87
S4	78.47	0.58	82.83	0.79
S5	59.39	0.68	55.65	0.47
S6	66.56	0.64	80.81	0.77
S7	86.34	0.53	87.38	0.85
S8	85.07	0.54	84.52	0.81
Average	74.02 ± 12.4	0.60 ± 0.1	79.20 ± 10.7	0.75 ± 0.1

**Table 6:** The SSVEP classification accuracy of FBCCA.

Subject	S1	S2	S3	S4	S5
Acc/%	100	97.22	95.83	98.61	100
Subject	S6	S7	S8	Avg	
Acc/%	97.22	100	100	98.61 ± 1.66	

In the proposed hybrid paradigm, the average SSVEP classification accuracy was 98.61 ± 1.61%, with the highest accuracy reaching 100%. All the above results demonstrate the effectiveness of the proposed hybrid paradigm for a group brain-controlled method.

Table 7 shows the average accuracy obtained from both EEG signals induced by precise hand movements and SSVEP responses. The highest accuracy was 93.69%, the lowest accuracy was 77.83%, and the average accuracy was 88.91 ± 5.06%.

**Table 7:** Statistical data for offline brain–computer interface decoding results.

Participant/ACC	MDFF-CNN	FBCCA	MDFF-CNN+ FBCCA
S1	73.51	100	86.76
S2	79.5	97.22	88.36
S3	89.42	95.83	92.63
S4	82.83	98.61	90.72
S5	55.65	100	77.83
S6	80.81	97.22	89.02
S7	87.38	100	93.69
S8	84.52	100	92.26
Average	79.20 ± 10.7	98.61 ± 1.66	88.91 ± 5.06

### 4.3 Online experimental analysis

The online experiments analyzed the practicality of the constructed group brain-controlled method for UAV swarm control. In the online experiment, eight participants were randomly assigned to five groups, and the correct rate of decoding the participants' brain-controlled intentions during two-participant collaboration was used as the criterion for evaluating the performance of the group brain-controlled system.

The online experiment results are shown in Table 8. The global accuracy of EEG signal decoding during the online experiment was 88.89 ± 1.96%. The highest accuracy was achieved by group G3, reaching 91.66%, while the lowest accuracy was obtained by group G1, reaching 86.11%. The online experimental results proved the feasibility of the proposed group brain-controlled method for UAV swarm control.

**Table 8:** Statistics of online BCI decoding results.

Test group	Number of correct commands	Online decoding accuracy
G1	32	88.89%
G2	32	88.89%
G3	33	91.66%
G4	31	86.11%
G5	32	88.89%
Mean ± STD	32 ± 0.7	88.89 ± 1.96%

## 5 Discussion

The results of this study demonstrate the effectiveness of the novel G-BCI system for UAV swarm control using a hybrid paradigm combining hand movements and visual evoked potentials. In total, three crucial factors were studied, including the hybrid group brain control paradigm, the corresponding MDFF-CNN decoding algorithms, and the brain–machine shared control strategy for UAV swarm control. The proposed paradigm combining precise hand movements and visual evoked potentials can increase the number of brain control commands and enhance the flexibility of complex task control. To fully use the features of the hybrid group brain control paradigm, an improved MDFF-CNN was used to decode EEG and EMG signals induced by precise hand movements, while FBCCA was employed for SSVEP signal decoding. For UAV swarm control, a shared control strategy integrating the G-BCI system and the leader–follower control method was constructed to realize flight movement and formation changes. Both offline and online experiments verified the effectiveness and feasibility of the proposed G-BCI system.

### 5.1 Mechanism and EEG signal decoding

Most G-BCI studies have focused on enhancing the overall signal-to-noise ratio and classification performance by optimizing static group coordination, yet it is generally constrained by a single EEG paradigm (Caiado and Ukolov, 2025). This results in inherent limitations in information dimensionality, command richness, and environmental adaptability, making it difficult to meet the demands of complex dynamic scenarios—such as the coordinated control of UAV swarms—which require simultaneous processing of discrete commands and continuous motion signals. Specifically, traditional systems face significant challenges in parallel decoding of multiple commands, real-time fusion of multimodal signals, and achieving highly robust control (Dai et al., 2025).

This study proposed a novel G-BCI system with a hybrid paradigm combining motion execution and SSVEPs. Moreover, multi-domain features from EEG and EMG signals were extracted using the MDFF-CNN model. Using the proposed G-BCI method, UAV swarms can realize both aerial motion control and formation switching. The proposed system achieved an overall accuracy of 88.89 ± 1.96% in the online test for completing complex tasks. These results demonstrate a significant advancement in developing a practical BCI system that can handle multiple commands.

Compared to existing single-paradigm G-BCI systems (Canal et al., 2023), the proposed hybrid G-BCI paradigm extends the brain control commands by combining both motion execution and SSVEPs, thereby improving command diversity and control flexibility for swarm control, especially for UAV swarm control. Specifically, six brain control commands based on hand movements can be recognized, and 30 brain–computer interaction commands can be realized by the G-BCI system. Moreover, compared to traditional EEG decoding methods (Galán et al., 2008) for precise hand movements, the incorporation of EEG and EMG signals and the MDFF-CNN model provides complementary physiological information for more robust intention recognition. Most importantly, when it comes to practical deployment, the existing shared control schemes suffer from the drawbacks of insufficient control commands and weak robustness. In contrast, the proposed system combines the G-BCI system with UAV swarm control, making it more suitable for completing control tasks with the benefits of multiple commands and flexible formation switching, which can greatly improve swarm control efficiency.

### 5.2 Limitations of the study and further research

Although this study has made progress in complex control tasks for group brain–computer interfaces (G-BCIs), it still faces three limitations: The lack of quantitative assessment of real-time inter-brain neural coupling, the limited capability of existing multimodal fusion methods to process complex heterogeneous signals, and the unvalidated generalizability of current experimental results across broader populations. Future research will, therefore, focus on developing more effective collaborative decoding algorithms to precisely characterize inter-brain dynamic interactions and enhance overall system performance. Concurrently, by further investigating neural mechanisms, optimizing algorithms, and expanding experimental validation, we aim to advance G-BCIs toward greater efficiency, robustness, and practical applicability.

## 6 Conclusion

In this study, a group brain-controlled method using a hybrid paradigm combining hand movement execution and visual evoked potentials was proposed. An improved MDFF-CNN model was constructed to decode the hybrid EEG and EMG signals associated with hand movement execution. The decoding accuracy of the MDFF-CNN model was 79.20 ± 10.7%, and the online experimental accuracy of the proposed system was 88.89 ± 1.96%, which effectively addressed the problem of insufficient brain control commands. Overall, the results demonstrated the effectiveness of the proposed group brain-controlled method. In future research, we will continue to improve our system, particularly by using the detection of error-related potentials as an arbitration mechanism and enhancing the adaptive capability of the MDFF-CNN algorithm, such as enabling its fusion weights to be learned through neural network training.

## Data Availability

The raw data supporting the conclusions of this article will be made available by the authors, without undue reservation.
